# Advancing Polymer Science and Energy Storage Solutions Through the Integration of Artificial Intelligence and Machine Learning: A Transformative Approach

**DOI:** 10.3390/polym17243267

**Published:** 2025-12-09

**Authors:** Turkan Kopac

**Affiliations:** Department of Chemistry, Zonguldak Bülent Ecevit University, 67100 Zonguldak, Türkiye; turkankopac@yahoo.com

**Keywords:** artificial intelligence, energy storage, machine learning, polymers, polymeric materials, porous polymers

## Abstract

Polymers play a pivotal role in advancing energy storage technologies because of their unique properties, including high conductivity, flexibility, and environmental stability. The integration of artificial intelligence (AI) and machine learning (ML) into polymer science has revolutionized the design, discovery, and optimization of polymeric materials, enabling the development of faster, cost-effective, and innovative solutions. This review explores the transformative impact of AI and ML in polymer science, focusing on their applications in polymer design, characterization, and energy storage. Key advancements include the accelerated discovery of materials, predictive modeling of polymer properties, and high-throughput screening of polymer candidates. This review highlights the strengths of AI and ML, including their ability to handle complex datasets, optimize multiproperty trade-offs, and uncover hidden relationships between structure and properties. However, challenges such as data limitations, model interpretability, and synthetic feasibility remain significant barriers to progress. The paper also identifies gaps in the literature, including the need for improved structural descriptors, expanded datasets, and the integration of physical principles into ML models. Future directions emphasize the development of sustainable polymers, generative design frameworks, and interdisciplinary collaboration to address pressing global challenges. By leveraging AI and ML, this work aims to accelerate the development of next-generation polymers for energy storage, fostering clean, efficient, and sustainable energy solutions.

## 1. Introduction

The increasing global demand for energy and the need for effective energy storage solutions have become increasingly urgent and are influenced by a combination of interrelated factors. The burgeoning population and accelerated urbanization, particularly in developing nations, have contributed to a relentless rise in energy consumption [1,2,3,4,5]. The transition toward renewable energy sources, including solar and wind power, presents significant challenges due to their inherent intermittency, thereby underscoring the critical importance of effective energy storage systems (ESSs) in securing a reliable power supply. Moreover, the imperative to combat climate change necessitates a marked reduction in reliance on fossil fuels, a transition facilitated by energy storage technologies that enable the seamless integration of renewable energy into existing power grids. These systems play a pivotal role in enhancing grid stability by effectively managing fluctuations in energy supply and demand, providing essential backup power during outages, and fostering energy independence through the utilization of locally generated resources. The increasing prevalence of electric vehicles further highlights the need for advanced energy storage solutions to adequately meet their power requirements. Additionally, the resilience provided by energy storage is vital in critical applications, ensuring an uninterrupted power supply in essential sectors such as healthcare and telecommunications. In summary, the strategic implementation of effective energy storage is crucial for achieving sustainable, reliable, and efficient energy solutions within the contemporary global landscape. It is integral in addressing energy demand, mitigating climate-related risks, and supporting the transition to renewable energy sources, thereby contributing significantly to the establishment of an environmentally sustainable global energy framework [6,7,8,9,10,11,12,13,14,15,16].

The performance of energy conversion and storage devices largely depends on the type of storage material used [17,18,19,20,21]. Various porous materials are currently being developed and studied as electrode materials for ESSs. These porous materials are preferred in many environmental and energy applications because of their ability to confine guest species on their external and internal surfaces and within their pore voids. Among the diverse range of porous materials, porous polymers have attracted significant interest due to their customizable pore structures and adjustable surface chemistry [8,22,23].

### 1.1. Exploring the Significance and Characteristics of Polymers in Energy Storage Solutions

**Figure 1:**
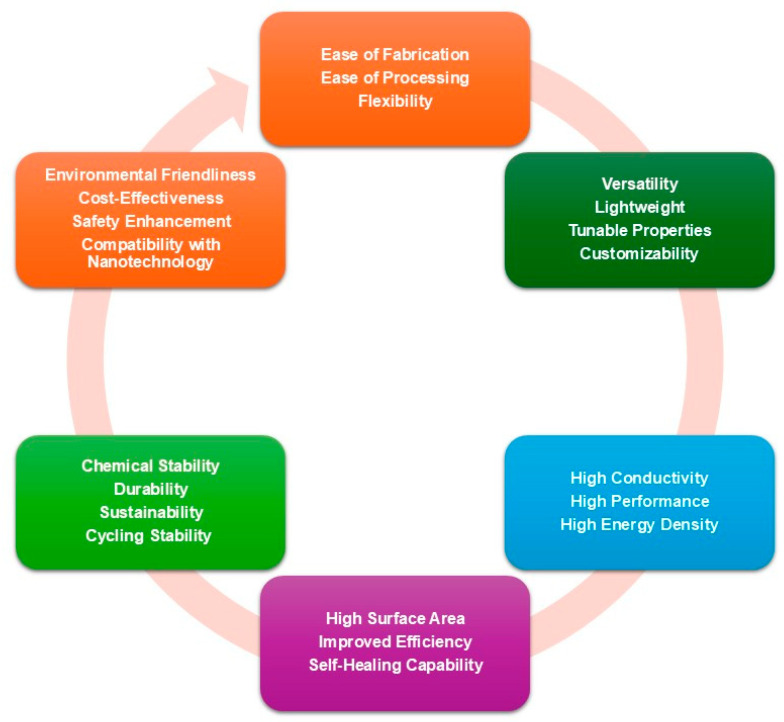
Key Properties of Polymers for Enhanced Energy Storage Solutions.

Polymers play a critical role in the field of energy storage because of their distinctive properties and inherent versatility. The importance of these materials becomes evident when we consider their key attributes, particularly in the context of energy storage applications [24,25,26]. These materials exhibit several essential characteristics, as depicted in Figure 1. Conducting polymers (CPs), such as polyaniline (PANI), polypyrrole (Ppy), and poly(3,4-ethylenedioxythiophene) (PEDOT), offer high conductivity, akin to that of metals, which facilitates efficient charge transport, making them excellent candidates for supercapacitors (SCs) and batteries [27,28,29,30]. The capacity of these materials to enhance charge storage in SCs is high, while polymeric composites enriched with carbon nanotubes (CNTs) or metal oxides significantly increase energy density, further optimizing battery and SC performance [28,31,32,33,34]. One of the remarkable advantages of polymers is their customizability; they can be chemically modified to enhance conductivity, ion transport, or mechanical properties. This adaptability extends to ease of processing, as polymers can be integrated into various manufacturing techniques, such as 3D and 4D printing. Safety is also a vital consideration, as solid polymer electrolytes (SPEs) replace flammable liquid electrolytes, enhancing battery safety. Additionally, their mechanical flexibility enables the development of portable and wearable energy devices, while their lightweight nature helps reduce the overall weight of ESSs. Polymers also possess high surface areas that improve energy storage efficiency and exhibit chemical stability (e.g., polymers such as Nafion^®^ and PEDOT) and mechanical strength, ensuring long-term performance. The ease of fabricating polymers into diverse forms, including films, fibers, and composites, via cost-effective methods contributes to their cost-effectiveness. Innovations in sustainable energy storage have led to the development of biodegradable and recyclable polymers, supporting eco-friendly solutions. Furthermore, these materials are nontoxic and can be synthesized with minimal environmental impact. The versatility of polymers allows for tailoring specific applications, such as SCs, batteries, and fuel cells, while their tunable properties can be adjusted through molecular design. Advanced polymers with self-healing capabilities increase device durability and lifespan. Finally, when combined with nanomaterials such as graphene and CNTs, polymers can create high-performance composites, improving efficiency by enabling faster charge/discharge rates and higher power density in ESSs. These characteristics of polymeric materials render them exceptionally versatile and effective for advancing energy storage applications, particularly within SCs, batteries, and fuel cells. Polymers are integral to the advancement of next-generation energy storage technologies, facilitating clean, efficient, and sustainable solutions to meet global energy demands [22,23].

**Figure 2:**
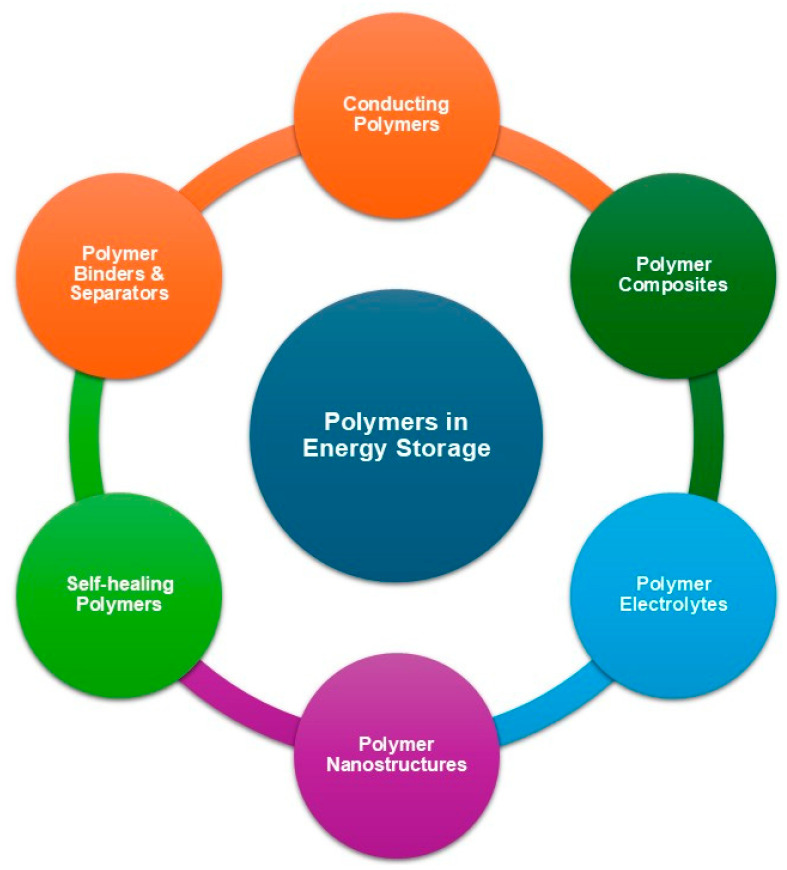
Exploring Advanced Polymers for Enhanced Energy Storage Solutions.

The polymers utilized in ESSs are outlined in Figure 2. The importance of polymers in energy storage applications is paramount, as they encompass a diverse array of materials specifically engineered to fulfill distinct functional requirements. CPs (PANI, Ppy, polythiophene (PTh), and PEDOT) possess metal-like conductivity achieved through structural modifications such as doping, making them ideal for SCs and batteries because of their high specific capacitance, conductivity, and electrochemical stability. Polymer composites, which combine CPs with carbon materials (e.g., graphene and CNTs) or metal oxides (e.g., RuO_2_ and MnO_2_), enhance the electrochemical performance by improving the conductivity, cycling stability, and energy density. In fuel cells, polymer-based electrolytes, such as Nafion^®^, are employed as proton-conducting membranes, providing flexibility and lightweight properties suitable for portable and wearable electronics. Other polymer matrices include sulfonated poly(ether ether ketone) and poly(benzimidazole). SPEs contribute to battery safety by replacing flammable liquid electrolytes, whereas polymer-based nanostructures, including nanofibers, nanotubes, and nanowires of CPs, increase the surface area and facilitate ion transport in SCs and batteries. Additionally, light-induced self-healing polymers, such as coumarin-functionalized polyisobutylene, enhance device durability, and polymer binders and separators optimize performance in lithium-ion and rechargeable batteries. Collectively, these polymers significantly contribute to the efficiency, safety, and adaptability of energy storage devices (ESDs), with applications spanning SCs, batteries, and fuel cells, owing to their high energy density, flexibility, and environmental stability [22,23].

### 1.2. Leveraging Machine Learning and Artificial Intelligence in Polymer Science: Innovations in Polymer Design and Characterization

ML and AI are becoming increasingly vital in the fields of polymer science, design, and characterization owing to their ability to overcome the limitations of traditional methodologies and expedite the discovery of new materials [35,36,37,38,39,40,41,42,43]. They accelerate the identification of structure–property relationships, which significantly shortens the time required for designing new polymers. Furthermore, AI facilitates inverse design, allowing researchers to define desired properties and backtrack to identify the molecular structures necessary to achieve them, thus enabling optimization across multiple properties. By providing data-driven insights that reveal connections and patterns beyond human intuition, ML enhances our understanding of polymers. It also improves performance by leveraging large datasets to predict polymer properties, processing conditions, and synthesis methods with high accuracy, thereby producing better-performing materials. Additionally, AI contributes to sustainability by assisting in the design of polymers that are more recyclable and environmentally friendly, which is crucial for addressing challenges in circular economies. The strength of ML in managing the stochastic nature of polymers, along with its application in high-throughput methodologies, further streamlines the experimental process. As it explores vast chemical spaces, ML identifies promising polymer candidates, ultimately reducing the cost and time needed for experimental synthesis. Techniques such as transfer learning allow ML to work even with limited datasets, addressing data limitations. The ability to extract features from complex 3D molecular structures into machine-readable formats ushers in a new era of analysis for the structural and topological characteristics of polymers. Moreover, advanced models like Variational Autoencoders (VAE) and Generative Adversarial Networks (GAN) and Reinforcement Learning (RL) enable the generation of novel polymer structures that meet specific criteria. By integrating ML with physical and chemical principles, researchers can increase prediction accuracy and solve complex optimization problems [35,36,37,38,39,40,41,42,43,44,45,46,47,48,49,50].

The combination of ML and AI is not only advancing polymer science but also revealing previously unexplored areas, enabling the discovery of new polymers and assisting researchers in overcoming cognitive blind spots. By integrating ML and AI methodologies, the field of polymer science is undergoing a revolution, streamlining rational design processes, optimizing the balance between various material properties, and identifying new avenues for research and innovation. In summary, ML and AI techniques are significantly accelerating the design, discovery, and characterization of polymeric materials while simultaneously enhancing the efficiency and creativity of these processes. This transformation is crucial for the development of advanced materials that are meticulously tailored to meet specific performance criteria [35,36,37,38,39,40,41,42,43,44,45,46,47,48,49,50].

### 1.3. Harnessing Artificial Intelligence and Machine Learning for Advancements in Polymer Energy Storage Solutions

AI and ML play pivotal roles in advancing polymers for energy storage by facilitating the discovery and design of materials with specific, tailored properties [51,52,53,54,55,56,57]. The expansive chemical space for polymers, which includes countless combinations of molecular structures, can be efficiently explored via ML, helping researchers identify promising candidates without undergoing exhaustive trial–and-error experiments. AI and ML techniques are also instrumental in screening and synthesizing polymer derivatives, allowing for the identification of optimal molecular structures that enhance energy storage performance. Furthermore, ML models can predict essential polymer properties, such as dielectric constants, bandgaps, and thermal stability, which are critical for applications in capacitors and batteries. They can also predict electrical properties, including breakdown strength and energy storage density, on the basis of specific molecular structures and trap parameters, significantly accelerating the design process. The ability to design inverse materials enables researchers to specify the desired properties (e.g., high energy density, low loss) and generate corresponding polymer structures efficiently. Additionally, by automating data analysis and modeling, AI and ML enhance research efficiency, enabling scientists to devote more time to innovative solutions for high-performance energy storage materials. Insights driven by AI can guide the optimization of molecular structures for greater energy storage performance, whereas high-throughput screening enables quick evaluation of extensive polymer libraries, reducing experimental costs and time. Moreover, AI helps uncover complex relationships between molecular chain segments and their impact on dielectric properties, facilitating precise modifications. By integrating data from simulations (density functional theory, DFT) and experiments, ML enhances the accuracy of predictions, and it can even optimize processing conditions to achieve the desired microstructures in ESDs. Ultimately, leveraging these advanced approaches, researchers can discover novel polymer materials that offer superior energy storage capabilities, leading to significant breakthroughs in flexible electronics, electric vehicles, and renewable energy systems. In summary, AI and ML are transformative tools that not only expedite the design and optimization process but also provide cost-effective and innovative solutions to meet the increasing demand for advanced energy storage materials, particularly in the development of next-generation polymer dielectrics with high energy storage capabilities [35,51,52,53,54,55,56,57,58,59,60,61,62,63,64].

### 1.4. Aim of This Contribution

The literature contains a limited number of publications and research studies focusing on the application of ML and AI in the fields of polymer science, polymer design, and characterization. Furthermore, there is a scarcity of research addressing these technologies, specifically for energy storage in polymers. Overall, the current body of work highlights a notable gap in studies exploring the integration of ML and AI techniques within polymer science and their implications for energy storage solutions.

The identified gaps in the literature related to ML and AI in polymer science, design, and characterization include the following:

1. The absence of large, high-quality datasets for specific polymer types—such as crosslinked networks and functional polymers such as shape memory polymers (SMPs) and vitrimers—restricts the accuracy and generalizability of ML models.
2. Existing structural representation methods, such as BigSMILES, struggle to encapsulate the complex topologies and morphological features of polymer networks, which hinders precise predictions.
3. Polymers recommended by AI may often be challenging to synthesize at scale, which limits their practical application.
4. The modeling of polymer composites, blends, and formulations remains a significant challenge because of intricate interactions and diverse transport mechanisms.
5. Limited or biased datasets may contribute to overfitting, compromising the reliability of predictions.
6. The integration of computational and experimental data, as well as the management of varying levels of fidelity, presents a technical obstacle.
7. Compared with conventional petroleum-based plastics, AI-designed sustainable polymers, including bioplastics, frequently face cost and scalability challenges.
8. The industrial-scale implementation of AI-driven polymer informatics is still in its early stages, facing hurdles in terms of technology transfer and acceptance.

There are also notable gaps in the literature regarding the applications of AI and ML in polymer energy storage.

1. There is a notable lack of high-quality datasets for specialized applications such as polymer energy storage, particularly concerning properties such as ionic conductivity, dielectric constants, and breakdown strength.
2. The mechanisms underlying charge traps and energy storage in polymers are highly intricate, which can lead AI models to oversimplify these phenomena.
3. AI models frequently encounter difficulties in generalizing across various polymer systems or in predicting the properties of novel polymers that are not adequately represented in training datasets.
4. Many AI models operate as “black boxes,” making it challenging to discern the mechanisms that drive their predictions.
5. Current AI models often fail to adequately consider long-term reliability and the environmental factors that impact polymer performance.
6. Predictions generated by AI and ML frameworks require extensive experimental validation, which can be both time-consuming and resource-intensive.
7. A limited body of research has focused on leveraging AI for the optimization of manufacturing processes, such as crosslinking and blending, to create scalable polymer-based ESSs.

Therefore, this review aims to address existing gaps in the literature by deriving key insights and proposing future research directions. This review explores publications and studies regarding the application of ML and AI within the fields of polymer science, polymer design, and characterization. Furthermore, the utilization of these technologies for energy storage in polymers will be examined. The primary focus will be to highlight the contributions of ML and AI in these domains while also identifying the strengths and weaknesses of their applications in polymer science and energy storage.

The integration of AI and ML in polymer science and energy storage offers significant opportunities for accelerating and enhancing material discovery and optimization, thereby promoting sustainability. AI holds transformative potential in polymer science, enabling rapid and innovative material discovery. It enhances predictive accuracy, streamlines design processes, and fosters the development of sustainable, high-performance polymers suitable for a wide array of applications.

While AI presents promising advancements for ESSs based on polymers, several challenges must be addressed, including data limitations, model interpretability, computational requirements, and the necessity for experimental validation. Addressing these challenges is essential to fully harness AI’s ability to create efficient, scalable, and sustainable energy storage materials.

AI can significantly improve the efficiency of ESSs in several critical ways. By accelerating material discovery, optimizing material properties, enhancing predictive modeling, and enabling real-time monitoring, AI plays a vital role in the development of high-performance, cost-effective, and sustainable energy storage solutions.

To address the prevailing gaps in the literature, future research endeavors must prioritize the expansion of datasets, enhancement of synthetic feasibility, integration of fundamental physical principles, optimization of manufacturing processes, and cultivation of interdisciplinary collaboration. These initiatives are critical for advancing the efficiency, scalability, and sustainability of AI-driven polymer energy storage research.

In light of the notable gaps in the current literature concerning the application of ML and AI in the fields of polymer science, polymer design, and characterization, as well as their utilization in ESSs involving polymers, this study aims to make several specific contributions, which are outlined as follows:

1. The study provides a detailed review of the literature on the application of ML and AI in polymer science, polymer design, and characterization, as well as their use in energy storage for polymers. The study identifies the methods utilized in AI and ML and evaluates their contributions to advancing polymer science and energy storage research.
2. The work systematically highlights gaps in the literature, such as data limitations, synthetic feasibility challenges, and the lack of integration of physical principles into AI models.
3. The manuscript is organized into two distinct parts. The first part focuses on the various ML and AI methodologies applied in polymer science, specifically addressing aspects of polymer design and characterization. In contrast, the second part delves into the unique applications of ML and AI techniques tailored for polymer energy storage solutions. Together, these sections offer a comprehensive exploration of how advanced computational approaches are influencing and enhancing the study and development of polymers.
4. The study provides a detailed analysis of the strengths and weaknesses of using AI and ML in polymer science and energy storage, offering insights into their transformative potential and limitations.
5. The work proposes actionable future directions to address the identified gaps, such as expanding datasets, integrating physical principles, optimizing manufacturing processes, and fostering interdisciplinary collaboration.
6. The study outlines a rigorous methodology for conducting literature reviews, including keyword searches, database selection, and exclusion criteria, ensuring a comprehensive and focused analysis.
7. By synthesizing existing knowledge and identifying areas for improvement, the work aims to accelerate advancements in polymer science and energy storage research. It emphasizes the transformative role of AI and ML in enabling efficient, sustainable, and innovative solutions.

The anticipated impact of this study can be encapsulated in several key areas. First, it advances the field of polymer science by contributing to the rational design and discovery of functional and sustainable polymers through the application of AI and ML. Second, it highlights the potential of AI and ML to optimize polymer-based ESSs, which is essential for addressing global energy challenges. Finally, the proposed future directions serve as a roadmap for researchers, guiding them in navigating existing limitations and fostering innovation within the field. Ultimately, this study not only expands the horizons of polymer science but also lays the groundwork for significant advancements in energy storage and research trajectories.

## 2. Methodology

This review is structured in two parts. The first part focuses on the literature that employs ML and AI techniques in the fields of polymer science, design, and characterization. The second part examines studies that apply these approaches specifically to polymer energy storage. The analysis encompasses the methods employed in AI and ML, as well as the contributions made by these techniques to polymer science, design, characterization, and energy storage research. The manuscript is organized as follows: the introduction highlights the significance and characteristics of polymers in energy storage solutions; addresses the types of polymers used in energy storage; and highlights the importance of integrating ML and AI in polymer science, design, and characterization. Additionally, the role of these technologies in advancing polymer energy storage has been explored. The aim of this contribution is also comprehensively outlined. The Section 2 outlines the methodology utilized in this study, detailing the key steps undertaken. Section 3 provides an overview of the literature, which is divided into two parts. The first part reviews the literature on ML approaches in polymer science, focusing on polymer design and characterization through AI and ML. This segment includes both a summary of review papers and a thorough analysis of published research papers. The second part examines AI and ML applications in polymer energy storage, also comprising an overview of review articles and an in-depth review of relevant research studies. Section 4 provides a comparative analysis of various ML methodologies. Section 5 presents a comprehensive analysis of the strengths and weaknesses associated with the use of AI and ML in polymer science, polymer discovery, and the design of functional and sustainable polymers, as well as in the context of polymer energy storage. Section 6 explores future directions, while Section 7 concludes the study.

The methodology involves a comprehensive review and evaluation of the literature on the applications of AI and ML techniques in polymer science, polymer design, characterization, and energy storage. The key steps in the following methodology are as follows:

1. Conducted an extensive search via keywords such as “polymers + polymer science + polymer design + polymer characterization + artificial intelligence (AI) + machine learning (ML)” and “polymers + energy storage + artificial intelligence (AI) + machine learning (ML)”.
2. Multiple databases were utilized for the literature search, including the Web of Science Core Collection, Scopus, and Google Scholar, which focused specifically on the Science Citation Index Expanded (SCIE) database.
3. Included articles published between 2016 and 2025 to ensure that the review covers recent advancements.
4. The exclusion criteria were as follows: proceedings papers, editorial materials, book chapters, and papers not published in English.
5. A rigorous manual examination of the collected articles was conducted to ensure relevance and quality.
6. The evaluation of the literature is organized into two distinct parts. Part 1 emphasizes the application of ML and AI in the field of polymer science, specifically addressing polymer design and characterization techniques. In contrast, Part 2 shifts the focus to the role of ML and AI in advancing polymer energy storage solutions. This structured analysis allows for a comprehensive understanding of how these cutting-edge technologies are transforming both the creation and functionality of polymers within different domains.
7. Evaluated the AI and ML methods utilized in the reviewed studies. The contributions of these methods to polymer design, characterization, and energy storage research were assessed.
8. Analyzed the strengths and weaknesses of using AI and ML in polymer science and energy storage.
9. Proposed actionable future directions on the basis of the gaps identified in the literature.

The methodology ensures a focused review of the literature, leveraging keyword searches, database selection, exclusion criteria, and manual examination to provide a comprehensive analysis of AI and ML applications in polymer science and energy storage. 

## 3. Overview of Literature Studies

Table 1 summarizes the literature on the application of ML and AI techniques in polymer science. It details the methods employed in these fields, particularly in the design and characterization of polymers through AI and ML, as well as the use of these technologies for energy storage in polymers. The first column identifies the specific methods applied in AI and ML, whereas the second column highlights their contributions to polymer design and characterization, along with their impact on polymer energy storage solutions.

### 3.1. Machine Learning Approaches in Polymer Science: Polymer Design and Characterization via AI-ML

#### 3.1.1. Analysis of Review Articles

By addressing challenges within a vast chemical landscape and the demand for tailored properties, Chen et al. [35] reviewed the utilization of ML in the design of organic molecules and polymers, emphasizing its transformative potential in materials design. This paper discusses the transition from traditional experimental methods to data-driven approaches, highlighting recent advancements in ML-guided design across the fields of biomedical, chemical, and materials science. It includes nine case studies that showcase applications, such as organic photovoltaics, polymer dielectrics, organic light-emitting diodes, polymeric solar cells, and high-energy materials. This study illustrates how ML can be integrated with other computational tools to increase the efficiency of material design. Overall, ML accelerates the design process and fosters innovation in polymers for various applications in energy, electronics, and materials science. Cencer et al. [36] explored the emerging discipline of polymer informatics, highlighting the application of ML in the design and prediction of the properties of polymeric materials. They also discuss the essential tools and databases needed for effective data sharing and analysis. Yan and Li [37] investigated the increasing role of ML in polymer discovery, detailing its potential benefits, processes, challenges, and the historical context surrounding its development within the field. Nasrin et al. [38] delve into the application of ML techniques in polymer additive manufacturing, addressing existing challenges, proposing solutions, and pinpointing areas for future research. Schuett et al. [39] provide a review of the integration of digital methods and automation in polymer science and engineering, underscoring their capacity to enhance the development of new polymer materials and processes. Tran et al. [40] explored the transformative influence of AI on accelerating polymer design and development. Their focus is on AI-driven advancements in polymer informatics and application-specific designs that cater to critical areas such as energy storage and sustainability. Cao et al. [43] evaluated the significant impact of ML within the field of polymer science, particularly its applications in autonomous synthesis, property prediction, and the creation of sustainable design frameworks aimed at enhancing material discovery and performance. Xie et al. [42] examined the integration of ML strategies in polymer science, emphasizing how these approaches improve the understanding, design, and application of polymer materials through data-driven methodologies. In their review article, Raza et al. [41] delved into the convergence of response surface methodology (RSM), ML, and AI to optimize the properties, production processes, and performance of polymeric nanocomposites. This article highlights the challenges, methodologies, and advancements in this interdisciplinary domain, emphasizing the importance of statistical and computational techniques in enhancing the mechanical, thermal, electrical, and electrochemical properties of polymer-based nanocomposites.

#### 3.1.2. An In-Depth Analysis of Scholarly Research Contributions

Dutta et al. [44] focused on an ML approach for the rapid characterization of SMPs, materials that change shape in response to stimuli such as heat, and are increasingly used in fields such as automotive, aerospace, and robotics. Their study combines video data analysis and scalable ML to create a predictive model for the recovery behavior of SMP laminates. Key contributions include a data-driven methodology for modeling SMP behavior; a standardized experimental protocol for collecting high-quality thermal and video data; and the development of predictive models with impressive accuracy, sensitivity, and specificity. This paper demonstrates the superior performance of an Ensemble Learner Network (ELN) over other models, such as Bayesian Ridge Regression (BRR) and Random Forest (RF). Findings are applied to simulate SMP-based soft robotics, highlighting the potential of SMPs in flexible systems. This study ultimately enhances the understanding and application of SMPs through ML for rapid material characterization.

Aldeghi and Coley [45] presented a novel graph-based representation and ML architecture, the weighted directed message passing neural network (wD-MPNN), to predict synthetic polymer properties. This approach captures essential features of polymers, such as chain architecture and stoichiometry, through a graph representation that includes “stochastic” edges to reflect the ensemble nature of polymers. The authors created a dataset of simulated electron affinity and ionization potential values for over 42,000 copolymers with varying monomer compositions, stoichiometries, and chain architectures and made it publicly available. The wD-MPNN outperforms traditional models in predicting properties, particularly for polymers with the same monomer composition but different architectures. The framework provides a basis for developing new ML techniques in polymer informatics, enhancing predictive accuracy and efficiency in the field.

Huang et al. [46] explored high thermal conductivity (TC) polymers via an interpretable ML framework combined with physical descriptors and high-throughput molecular dynamics simulations (MDSs). This study addresses the inefficiencies of traditional trial–and–error approaches to polymer design by introducing a data-driven method. Their framework reduces 320 initial physical descriptors to 20 optimized descriptors, achieving a prediction accuracy (R^2^) over 0.80, which outperforms traditional graph descriptors. They identified 107 promising polymers with TCs greater than 20.00 W/mK, 29 of which had a synthetic accessibility score of 3.00 or lower, indicating the feasibility of experimental synthesis. This study highlights key physical factors impacting TC, such as the cross-sectional area and dihedral stiffness, through SHapley Additive exPlanations (SHAP) analysis and emphasizes the role of π-conjugated structures in enhancing the thermal properties. Additionally, the authors derived mathematical formulas for TC prediction, allowing rapid screening without relying solely on ML models. They investigated thermal transport mechanisms and revealed that strong intrachain interactions improve thermal transport in amorphous states. This work provides a systematic approach to designing high-TC polymers, bridging theoretical predictions and experimental realizations while advancing polymer informatics.

Shah et al. [47] explored the application of ML techniques to optimize the bead foam extrusion process for polylactic acid (PLA), a biodegradable polymer. This study highlights the limitations of traditional empirical methods in predicting melt pressure during underwater granulation (UWG) and bead foam density, with a focus on the complex relationships between processing parameters, including temperature, screw speed, injector pressure, and CO_2_ content. Key contributions include the innovative use of models such as RF, Decision Tree (DT), Gradient Boosting (GB), Least Absolute Shrinkage and Selection Operator (LASSO), Support Vector Regressor (SVR), with the RF model achieving impressive R^2^ scores of 0.96 for melt pressure in the UWG and the DT model achieving an R^2^ score of 0.83 for predicting bead foam density. This finding highlights the ability of ML to uncover intricate parameter interactions and identify key factors affecting both properties, facilitating targeted process optimization. This study identifies influential parameters for melt pressure (e.g., CO_2_ content, injector pressure, and temperature in the B-extruder) and bead density (e.g., CO_2_ content, die plate temperature, and melt pressure in the B-extruder). Additionally, correlation analysis offered further insights into processing dynamics, promoting sustainability and efficiency in PLA foam manufacturing. The successful application of ML in this study also opens avenues for similar approaches in other polymer systems, reinforcing the potential of ML to enhance material design and processing efficiency while advocating for more sustainable alternatives to traditional petroleum-based plastics.

Shah et al. [49] focused on optimizing low-density polyamide 12 (PA-12) foams via advanced AI and ML techniques. This study introduces a Bayesian optimization (BO) framework integrated with active learning (AL) to minimize the foam density from 900 kg/m^3^ to as low as 50 kg/m^3^, thereby reducing the number of experimental trials. It employs an inverse design to predict optimal processing parameters, emphasizing the critical role of temperature and the flexibility of pressure in foam density. Research has also integrated various ML models to analyze process–property relationships and optimize foam morphology through scanning electron microscopy (SEM) analysis. This contributes to sustainability by minimizing material waste and energy use. These findings have significant implications for various industries, advancing the potential of AI and ML in polymer design and manufacturing.

Advincula et al. [48] investigated the application of AI and ML in optimizing the polymerization and copolymerization processes. This study presents innovative protocols designed to enhance the synthesis and manufacturing of polymers through AI/ML techniques, specifically employing autonomous continuous flow chemistry reactors that utilize real-time ML feedback for precise control. It also explores the integration of digital twins, advanced spectroscopic tools, and computational models to refine classical theories, such as the Mayo–Lewis equation (MLE), and enables the design of polymers with targeted properties. Among its key contributions, this study highlights AI/ML-driven polymerization, which enhances synthesis efficiency by optimizing reaction parameters (e.g., temperature, pressure, and monomer ratios) via real-time monitoring. Additionally, it introduces the development of digital twins—virtual replicas of polymerization systems—which help predict and optimize reaction outcomes while deepening the understanding of polymerization mechanisms. The research also refines classical models, notably revising the MLE with ML to improve predictions of copolymer composition and sequence distribution. Furthermore, it integrates advanced tools, combining spectroscopic and microscopic characterization techniques with ML for real-time analysis across various reaction setups. Through data-driven discovery, the study employs large language models (LLMs) to extract synthesis protocols from the literature, facilitating reaction optimization. Ultimately, this study highlights the design of functional polymers by creating tailored homopolymers and copolymers while also exploring AI/ML methods for the development of new materials. Overall, the paper establishes a foundation for the advancement of autonomous AI-driven laboratories, fostering innovation in polymer science and connecting theoretical modeling with experimental synthesis.

Wang et al. [50] introduced polybots, AI-driven autonomous platforms for processing electronic polymers, specifically thin films of poly(3,4-ethylenedioxythiophene) doped with poly(4-styrenesulfonate) (PEDOT:PSS). This study focuses on the efficient creation of high-conductivity, low-defect thin films, which are essential for electronics and energy devices. Polybot uses advanced ML algorithms, statistical methods, robotic systems, and importance-guided BO to explore a complex 7-dimensional parameter space, allowing for simultaneous optimization of multiple objectives such as conductivity and defect minimization. The platform successfully fabricates transparent conductive thin films with conductivities exceeding 4500 S/cm, rivaling state-of-the-art levels. This research offers insights into how experimental parameters influence film properties and identifies structural features that enhance performance via methods such as SHAP and cryogenic electron microscopy (cryo-EM). Overall, this study demonstrates the potential of AI-driven automation to transform electronic polymer manufacturing, paving the way for scalable, efficient materials discovery and production. This work sets a new benchmark for the discovery of innovative materials.

### 3.2. Artificial Intelligence Machine Learning Approaches for Polymers’ Energy Storage

#### 3.2.1. Comprehensive Review Summary

Wang et al. [57] presented a review on the role of AI, particularly focusing on ML and deep learning (DL), in the advancement of solid-state battery (SSB) technology. This review emphasizes how AI accelerates material screening, predicts battery performance, and addresses the challenges associated with SSB development. Gao and Lu [52] provide a comprehensive overview of the application of ML technologies in the development and management of energy storage devices (ESDs) and ESSs. They underscore the increasing importance of ML in tackling challenges such as state estimation, lifetime prediction, fault diagnosis, property analysis, modeling, design, and optimization for ESDs and ESSs. Shen et al. [54] reviewed the application of ML in the research and development of energy storage materials, specifically emphasizing dielectric capacitors (DCs) and lithium-ion batteries (LIBs). They highlight the transformative impact of ML in accelerating the discovery, design, and optimization of energy storage materials. Additionally, Shi et al. [51] provide a thorough review of advancements in wearable electronics and photonics, focusing on their integration with AI and the Internet of Things (IoT). They highlight significant progress in materials, transducing mechanisms, structural configurations, and various applications, including healthcare monitoring, human–machine interfaces, robotics, and flexible displays. Finegan et al. [53] offered insights into the utilization of ML techniques to enhance the characterization of LIB electrodes. They emphasize the importance of material characterization in understanding the structure–function relationships of electrodes and in overcoming performance limitations. Meng et al. [55] reviewed advancements in polymer dielectrics known for their high energy storage performance, focusing on the design and regulation of electric charge trap structures. They highlight the challenges associated with improving the energy storage performance of polymer DCs, particularly under high electric fields and elevated temperatures, and present a systematic overview of recent progress made in addressing these issues. Sun et al. [56] discussed recent developments in smart flexible sensing systems powered by AI, emphasizing the integration of ML and artificial synapses to enhance data processing and application capabilities.

#### 3.2.2. An In-Depth Analysis of Scholarly Research Contributions

Mannodi-Kanakkithodi et al. [58] developed an ML framework to accelerate the design of polymer dielectrics, which are crucial for electrical insulation, capacitive energy storage, organic photovoltaics, and flexible electronics. The traditional design process is inefficient, time-consuming, and computationally expensive and relies on high-throughput experiments or simulations. This paper introduces an efficient ML-based approach using genetic algorithms. Key contributions include a robust dataset of 284 symmetry-unique 4-block polymers built from seven chemical building blocks whose properties, such as bandgap and dielectric constants, are calculated via first-principles computations. The authors introduced a numerical representation (fingerprinting) of these polymers to correlate their structures with their properties. An ML model, Kernel Ridge Regression (KRR), was created to predict properties with over 90% accuracy compared with first-principles calculations. They implemented a genetic algorithm for designing polymers with desired dielectric constants and band gaps, significantly reducing computational effort by bypassing exhaustive enumeration. The framework also extends to longer repeat units (6-block and 8-block polymers), confirming its scalability. While focused on polymer dielectrics, the methodology is adaptable to other material classes. This novel approach enables rapid identification of promising materials for synthesis and testing, representing a significant advance in materials design.

Feng et al. [60] explored the use of ML in designing and optimizing polymer nanocomposites for energy storage. The study predicts the maximum energy storage density and enhances composite structures via a dataset of 1254 data points from 233 studies. Three ML algorithms—RF, Support Vector Machine (SVM), and Neural Network (NN)—were employed, with the RF model achieving the highest accuracy (91.9%) when visual image data (SEM images) were incorporated. The models were validated by creating composite films in the laboratory, which aligned with predictions. Additionally, the influence of various descriptors (e.g., matrix properties, filler content, filler shape, and coating) on energy storage performance was analyzed, and optimal filler structures, such as the coating thickness for TiO_2_, SiO_2_, and Al_2_O_3,_ were identified. Overall, this research highlights how ML can streamline the design process for high-energy-density composites for energy storage, reducing time and material waste.

Kern et al. [59] explored innovative polymer designs for energy storage capacitors, particularly in challenging environments characterized by high temperatures and electric fields. This research uses a combination of ML and genetic algorithms (GA) to effectively identify polymers that exhibit desirable properties, such as high energy density and thermal stability. Among the key contributions of the study are the enhancement of the GA framework to include a broader range of property criteria, such as the bandgap and dielectric constant, and the introduction of a clamping fitness function that allows for the management of conflicting properties. Additionally, the study incorporates strategies aimed at ensuring both chemical diversity and synthetic feasibility. A user-friendly interface was developed to enable the customization of polymer designs by specifying functional groups. The researchers successfully designed over 50,000 hypothetical polymers, leading to the identification of 23 candidates likely to be synthesizable. Four notable examples emerged from this process, and the modified GA code was made publicly available on GitHub. While the primary focus is on high-temperature dielectrics, the methodologies outlined in this study are applicable across various polymer applications, including membranes for batteries, fuel cells, gas separation, and recyclable plastics. Overall, this research provides a valuable framework for accelerating the discovery of high-performance materials for energy storage and other uses. Mannodi-Kanakkithodi et al. [58] and Kern et al. [59] investigate polymer dielectrics for energy storage using differing methodologies. Mannodi-Kanakkithodi et al. [58] employs a predict-and-screen approach, utilizing a dataset of 284 unique 4-block polymers to predict properties like dielectric constants and band gaps through KRR, achieving over 90% accuracy. However, this method is limited by dataset quality and lacks exploration of new polymer structures. In contrast, Kern et al. [59] use an inverse design approach with GA to create polymers that satisfy specific performance criteria, such as high energy density and thermal stability for energy storage capacitors, ensuring synthetic feasibility and chemical diversity. While effective for multiobjective optimization, it has high computational demands and requires extensive validation.

Saco et al. [61] explored optimizing Proton Exchange Membrane Fuel Cells (PEMFC) performance via ML algorithms. While PEMFCs are efficient and cost effective, their performance can be enhanced with advanced technologies. This study examines the effects of different humidity levels on PEMFC performance and compares ML models—Support Vector Machine Regression (SVMR), Linear Regression (LR), and K-Nearest Neighbor (KNN)—to identify the best predictive model. The results indicate that the LR model achieves the highest accuracy with the lowest error rate (RMSE of 0.0034). Additionally, it analyzes various flow channel designs (e.g., serpentine and zigzag) and finds that machine learning predictions align closely with numerical simulations. The authors suggest extending the research to DL and including more parameters such as oxygen levels, water content, and various cell designs. Overall, the paper demonstrates how ML can effectively enhance PEMFC design and performance.

Nistane et al. [64] explored the development of sustainable, high-performance polymer membranes for the separation of toluene and heptane via a combination of simulations, experiments, and ML. They enhance diffusivity prediction through advanced multitask and physics-enforced models that leverage both experimental and simulated data, improving accuracy in data-scarce situations. This study identifies polyvinyl chloride (PVC) as the optimal membrane for this separation, validating findings with ML-predicted trade-off plots. Additionally, they screened 13,000 known and 8 million virtual polymers to suggest halogen-free, environmentally friendly alternatives. Extensive trade-off plots for various polymers provide a rapid framework for membrane design. Overall, this research combines data-driven approaches and physical principles to advance membrane technology and address the limitations of traditional methods.

Chen et al. [62] explored the development of polymer nanocomposites using carbon-based additives such as CNTs and short-cut carbon fibers (CFs) for energy applications. This study combines experimental evaluations with a hybrid ML framework to increase the electrical conductivity and self-heating performance of polydimethylsiloxane (PDMS)-based composites. The key findings include that longer CFs (6 mm) significantly improve the conductivity (up to 1.8 S/m) and thermal response, with better performance observed under various input voltages (8–12 V). A hybrid ML framework utilizing Random Forest Regression (RFR) and Support Vector Regression (SVR) achieved high predictive accuracy (R^2^ > 0.95), identifying optimal design configurations. The results indicate that the CF length and input voltage are crucial for thermal-electrical performance. This research moves beyond traditional methods by integrating data-driven insights with physical interpretations, offering a framework for designing advanced polymer composites with enhanced electrothermal properties for applications in energy systems, deicing, and wearable technologies.

Maeda et al. [63] explored the use of ML to discover liquid crystalline polymers (LCPs) with high TC, which are essential for efficient heat dissipation in next-generation power electronics. The authors developed an ML model with over 96% accuracy to predict a polymer’s formation of a liquid crystalline phase on the basis of its chemical structure. They conducted a high-throughput screening of 115,536 polyimides, identifying 10,825 potential candidates. Six samples were synthesized, all of which formed smectic liquid crystalline phases, with TCs between 0.722 and 1.26 W/mK, outperforming commercial amorphous polyimides. This study highlights the importance of molecular orientation and chain rigidity in determining thermal conductivity, highlighting significant advancements in data-driven polymer material design and demonstrating the potential to accelerate the discovery of advanced materials.

## 4. Comparative Analysis of Different Methodologies

A comparative analysis of various ML models employed in the studies highlights several important insights. Notably, prediction accuracy, typically measured by the coefficient of determination (R^2^), demonstrates that graph neural networks (GNNs) and neural networks (NNs) often outperform others in predicting complex molecular properties, due to their ability to model intricate relationships. For instance, research by Aldeghi and Coley [45] has shown GNNs achieving R^2^ values greater than 0.90 when predicting polymer properties. RF models, although simpler, also deliver high accuracy, with R^2^ values above 0.91 in specific tasks like energy storage density prediction and processing parameter optimization, as noted by Feng et al. [60] and Shah et al. [47], respectively. However, computational cost is a factor; GNNs and NNs are more resource-intensive given their reliance on large datasets and complex architectures, making them less suited for scenarios with limited data or resources. In contrast, RF and BO models present more computational efficiency, making them suitable for tasks with limited data or resources. The context-dependent advantages of these models are notable: GNNs are ideal for detailed molecular structure-property relationships, particularly in stochastic systems like copolymers, while RF models are more effective for smaller datasets and simpler relationships, such as those found in processing parameter optimization. BO is especially powerful for optimization tasks, allowing for fewer experimental trials, such as minimizing foam density [49] or maximizing energy storage density, with fewer experimental trials, and SVMs, although effective for classification tasks with small datasets, can struggle with scalability. Lastly, NNs are suitable for highly nonlinear relationships but require careful tuning to minimize the risk of overfitting. The development of hybrid models that leverage the strengths of various ML techniques—such as combining GNNs for feature extraction with RF for prediction—has the potential to improve both prediction accuracy and computational efficiency in polymer property prediction. Additionally, evaluating the specific nature of the polymer property prediction tasks and considering available datasets can help select the most suitable ML model for these studies.

A comparative analysis of representation methods for specific polymer classes, such as branched or cross-linked networks, highlights the strengths of graph-based approaches over sequence-based ones. While methods like BigSMILES struggle with complex topologies, graph-based representations, such as wD-MPNN, effectively capture structural nuances, including chain architecture and stoichiometry, particularly for stochastic polymer ensembles, as shown by Aldeghi and Coley [45]. Sequence-based methods work for simpler systems but are inadequate for intricate polymers. Graph-based methods effectively capture intricate details in branched or cross-linked polymers and offer high accuracy in property prediction, though they may demand more computational resources. Future research should focus on integrating these methods or creating hybrids to address their limitations. Advanced descriptors, such as G-BigSMILES, could improve the representation of polydispersity and long-chain properties, thereby enhancing predictions for complex polymer systems.

Dutta et al. [44] and Nistane et al. [64] showcase distinct approaches in polymer science—Dutta et al. [44] focus on a data-driven method using ML models like BRR and RF to predict the recovery behavior of SMP through video data analysis. This approach is user-friendly and allows for rapid material characterization but lacks physical insights and relies heavily on high-quality data. Conversely, Nistane et al. [64] adopt a physics-informed, data-driven approach for designing polymer membranes, using physics-enforced neural networks (PENN) and data augmentation to enhance prediction accuracy. This method integrates physical principles, improving interpretability and generalizability while being robust in utilizing both experimental data and simulations. However, it requires deep knowledge of polymer physics and can be computationally intensive. The data-driven approach is ideal when high-quality experimental data is abundant, while the physics-informed method excels in scenarios with limited data, such as complex systems needing high accuracy and interpretability. The future of polymer science may benefit from hybrid models that combine both methodologies, leveraging transfer learning and collaborations between data scientists and polymer physicists to create innovative materials for sustainability and performance.

AI/ML models in polymer science encounter limitations due to data quality and availability. These models rely on large, high-quality datasets, which are often scarce in niche areas like energy storage and novel materials such as SMPs. This can lead to overfitting and unreliable predictions. Expanding datasets through sharing initiatives, synthetic data generation, and collaboration is essential. Additionally, the computational costs of advanced models restrict accessibility for smaller research groups, highlighting the need for lightweight models, cloud computing, and optimized algorithms. Synthetic feasibility is also an issue, as AI/ML may propose polymer structures difficult to synthesize. Employing constraints like VFS can help ensure manufacturability. Modeling polymer composites and blends is complex due to intricate interactions, often leading to oversimplifications. Multiscale modeling, combining molecular simulations and performance predictions, may improve effectiveness in these complex systems. Ethical concerns include proprietary algorithms limiting access to knowledge and the environmental impact of computational models. Promoting open-source tools and energy-efficient practices can address these issues. While AI-designed sustainable polymers like bioplastics offer innovative solutions, economic viability remains a challenge compared to petroleum-based plastics. Combining data-driven and physics-informed approaches could tackle these challenges. Creating hybrid models that leverage both methodologies and fostering interdisciplinary collaboration can drive innovation while addressing ethical and economic issues in polymer science.

Model interpretability is essential in polymer design, as unclear predictions can hinder understanding and delay rational development. While DL models like wD-MPNNs can accurately predict polymer properties, their lack of transparency limits insight into structure-property relationships, as noted by Aldeghi and Coley [45]. Physical descriptors such as cross-sectional area and dihedral stiffness are crucial for determining thermal conductivity, as highlighted by Huang et al. [46], but without techniques like SHAP, models remain “black boxes.” This obscurity poses risks in safety-critical areas like energy storage. Employing XAI techniques, like SHAP and feature importance analysis, can enhance trust and understanding in model predictions. Overfitting is a significant challenge in polymer science, affecting DNNs, generative models, and Monte Carlo methods. DNNs may memorize limited training data, leading to poor generalization, while generative models like VAEs and GANs, may yield results too similar to their training data, limiting innovation. Monte Carlo methods can also produce biased predictions with insufficient data. Solutions include regularization, data augmentation, transfer learning, and cross-validation to mitigate overfitting risks.

Research studies can be categorized based on primary objectives—property prediction, inverse design, and process optimization, as each goal requires different ML paradigms, methodologies, and evaluation metrics. Property prediction in polymers aims to estimate characteristics like dielectric constants, ionic conductivity, and thermal stability based on molecular structures or experimental conditions. Supervised learning techniques, such as RF, SVR, and KRR, are commonly utilized for these predictions. DL methods, including GNNs and Multilayer Perceptrons, address more complex aspects such as thermal conductivity and charge transport behavior. Studies, like those by Huang et al. [46] on high thermal conductivity polymers and Mannodi-Kanakkithodi et al. [58] on dielectric constants with KRR, show higher accuracy rates compared to first-principles calculations. Challenges include data scarcity for niche properties, a risk of overfitting, and a lack of interpretability in complex models. Common evaluation metrics are the coefficient of determination (R^2^), Mean Squared Error (MSE), and Root Mean Square Error (RMSE). Inverse design involves defining desired polymer properties and using ML to identify molecular structures or compositions that can achieve them. Generative models like VAEs and GANs are used to generate new polymer structures, while optimization algorithms such as GA and BO efficiently explore chemical spaces. For instance, Kern et al. [59] employed GA to design energy storage polymers, identifying 23 synthesizable candidates. Mannodi-Kanakkithodi et al. [58] also used GA to design polymer dielectrics with specific properties without exhaustive searches. Challenges remain, including synthetic feasibility, limited generalization, and high computational costs. Evaluation metrics focus on the success rate of synthesizable polymers, the diversity and novelty of generated structures, and the correlation between predicted and experimentally validated properties. The objective of process optimization is to enhance the properties of polymers, reduce costs, and improve scalability. To achieve this, various ML paradigms are utilized. Supervised learning techniques, including regression models like GB and DTR, help predict process outcomes effectively. In addition, active learning methods such as BO frameworks are employed to reduce the number of experimental trials while fine-tuning process parameters. For instance, Shah et al. [47] applied ML to optimize the bead foam extrusion process for PLA, resulting in high prediction accuracy for melt pressure and bead foam density. Similarly, Advincula et al. [48] harnessed AI-driven continuous flow chemistry reactors to optimize polymerization processes, utilizing real-time ML feedback for precise control. However, challenges remain, including modeling the complex interactions found in polymer blends and composites, dealing with limited datasets for specialized manufacturing processes, and navigating high-dimensional search spaces for process parameters. Success in these endeavors can be measured through metrics such as the reduction in experimental trials, improvements in process efficiency and scalability, and the accuracy of predictions regarding the relationships between process variables and material properties.

## 5. Strengths and Weaknesses

### 5.1. Strengths and Weaknesses of Using Artificial Intelligence and Machine Learning in Polymer Science, Polymer Discovery, and the Design of Functional and Sustainable Polymers

The strengths of using AI and ML in polymer science are substantial and transformative. Firstly, these technologies accelerate the discovery process by significantly shortening the time required for designing and developing new polymers, automating property predictions and material screening. They are adept at handling complex data, allowing ML models to process large datasets and reveal intricate nonlinear relationships between polymer structures and their properties. Additionally, AI-powered workflows can efficiently explore vast chemical and configurational spaces, leading to the discovery of novel polymers that might not be intuitively identified by humans. Advanced fingerprinting methods enable feature extraction from molecular structures, contributing to more accurate predictions of polymer behavior. Modern ML techniques, including multitask learning and GNNs, further enhance the accuracy and interpretability of predictions across diverse datasets. Moreover, ML frameworks, such as Bayesian models and reinforcement learning, facilitate inverse design, generating new polymer structures tailored to specific requirements. The use of VFS allows for the creation of synthetically feasible polymers by leveraging known reaction templates and commercially available monomers. By combining high-fidelity experimental data with low-fidelity simulation data, multifidelity learning improves model robustness and generalizability. Incorporating known physical principles into ML models ensures adherence to scientific laws, thus enhancing prediction accuracy and interpretability. AI also democratizes the expertise in polymer design, making knowledge accessible to nonexperts and fostering cross-disciplinary innovation. Furthermore, AI contributes to sustainability efforts by aiding in the design of biodegradable and chemically recyclable polymers, addressing critical issues like plastic pollution. Ultimately, the cost efficiency achieved through reduced experimental trials minimizes expenses related to manpower, materials, and time, underscoring the value of AI and ML in advancing polymer science.

The application of AI and ML in polymer science faces several notable limitations. First and foremost, there is a significant issue with data availability; ML models require large, high-quality datasets, which are often scarce for specific types of polymers, particularly crosslinked networks and functional polymers like SMPs and vitrimers. Additionally, existing fingerprinting methods struggle to adequately capture the complex topologies and morphological features present in polymer networks. Another challenge lies in synthetic feasibility; many polymers suggested by AI may be difficult or impractical to synthesize on a large scale, hindering their real-world applicability. The inherent complexity of polymer systems, which include composites, blends, and formulations, poses challenges for accurate modeling through current AI techniques. Predicting the behavior of polymers in complex mixtures, whether liquid or gas, adds another layer of difficulty due to the high-dimensional search spaces and various transport mechanisms involved. Moreover, some advanced ML methods, such as Monte Carlo algorithms and DNNS, are computationally intensive and time-consuming. There is also the risk of overfitting, particularly when limited or biased datasets are utilized, which undermines the reliability of predictions. Many ML models function as “black boxes,” lacking a conceptual understanding of the phenomena they aim to predict, thus complicating global optimization processes. The reliance on datasets that may not conform to a Gaussian distribution negatively impacts model accuracy, and experimental validation is often required for AI predictions, which can be both time-consuming and resource-intensive. Furthermore, biases in training data, stemming from the dominance of certain polymer classes, can lead to missed opportunities for novel discoveries. Finally, integrating computational and experimental data while addressing varying fidelity levels presents technical challenges, and the economic viability of AI-designed sustainable polymers, such as bioplastics, often pales in comparison to traditional petroleum-based plastics. Consequently, the industrial-scale implementation of AI-driven polymer informatics is still in its early stages, facing persistent obstacles in technology transfer and acceptance.

Integrating synthetic accessibility scores and retrosynthesis algorithms into the ML design loop is essential for aligning in silico discoveries with practical polymer fabrication. By evaluating monomer availability, reaction complexity, and cost, synthetic accessibility can enhance ML model optimization. Techniques like symbolic regression and BO prioritize polymers that are both high-performing and easily fabricated. Retrosynthesis algorithms, such as the Polymer Retrosynthesis Algorithm mentioned in Kern et al. [59], can trace back from desired polymer properties to viable synthetic pathways, utilizing reaction templates and databases of available monomers. Generative models like VAEs and GANs can be trained to produce polymer structures that meet performance and feasibility criteria. Real-time experimental feedback further refines these models, with platforms such as Polybots introduced by Wang et al. [50] aiding the iterative process. Incorporating synthetic accessibility into polymer discovery process offers several significant benefits that enhance both feasibility and efficiency. This approach emphasizes polymers that are scalable for manufacturing, enhancing cost efficiency and reducing the resources needed for validation. Integrating retrosynthesis algorithms accelerates the identification of viable polymer candidates, facilitating faster transitions to practical applications and driving innovation in polymer development. The challenges in polymer synthesis are largely linked to issues of data availability, the complexity of polymer synthesis, and high computational costs. High-quality datasets with synthetic accessibility scores and retrosynthesis pathways are limited, affecting the accuracy of ML models. The complexity of polymer reactions also complicates the development of universal retrosynthesis algorithms. Future research should focus on creating standardized synthetic accessibility scoring and expanding polymer-specific retrosynthesis databases.

### 5.2. Strengths and Weaknesses of Using Artificial Intelligence and Machine Learning for Polymers Energy Storage

The application of AI and ML in the domain of energy storage for polymers offers numerous advantages that significantly enhance the development process. Firstly, these technologies enable efficient material screening, allowing for the accelerated discovery of high-performance polymer electrolytes by analyzing extensive datasets and predicting critical properties like ionic conductivity and mechanical strength. By rapidly identifying suitable polymers, AI and ML reduce the time and resources needed for experimental trials. Additionally, they excel in data analysis, uncovering patterns in complex experimental datasets, including trap characterization and electrical property data. High-throughput analysis further enhances this capability, facilitating the screening of thousands of polymer candidates quickly and cost-effectively. Moreover, AI optimizes polymer compositions and structures to improve performance metrics such as ionic conductivity, thermal stability, and electrochemical stability, thereby boosting energy storage efficiency. Predictive modeling allows researchers to foresee the behavior of polymer-based electrolytes under varying conditions, which assists in designing more robust ESSs. Furthermore, ML enhances the accuracy of simulations, including phase-field modeling, to better predict breakdown processes and charge transport behavior. The integration of experimental data with theoretical models increases the precision of predictions, guiding the synthesis of innovative polymer materials tailored for specific applications, whether in high-temperature or high-voltage environments. Ultimately, by minimizing the need for extensive physical testing, AI and ML contribute to significant cost efficiencies in research and development, streamlining the advancement of energy storage technologies.

The application of AI and ML in energy storage for polymers presents several weaknesses that must be considered. One major challenge is the reliance on high-quality and comprehensive datasets for training; however, such data may be scarce and inconsistent in specialized areas like polymer energy storage, which can hinder model accuracy. Additionally, the complex and diverse structures of polymers make it difficult to accurately model their behavior, as the underlying mechanisms of charge traps and energy storage are intricate and may be oversimplified by AI models. Furthermore, AI models designed for one polymer system may lack portability, necessitating extensive retraining to apply them to other systems. Interpretability remains a significant concern, particularly with DL models that often operate as “black boxes,” obscuring the mechanisms behind their predictions. The computational costs associated with training and executing ML models can also be substantial, especially for large-scale datasets. Moreover, while AI and ML can generate predictions, these require experimental validation, which can be both time-consuming and resource-intensive. Many AI models may also exhibit limited generalization capabilities, struggling to predict properties for novel polymers that were not included in the training set. Lastly, there is a limited scope in current AI models, as they often fail to adequately address long-term reliability and environmental factors that impact polymer performance.

AI faces challenges in predicting the long-term reliability of polymers in energy storage devices due to factors like operational stress, environmental factors, and degradation over time. Integrating accelerated aging data with ML models can improve predictions by simulating long-term degradation in controlled environments like elevated temperatures, humidity, and mechanical stress, thus providing valuable training data. This dual approach allows ML models to effectively learn degradation patterns. Multifidelity modeling enhances material science predictions by combining short-term simulations with long-term degradation models. High-fidelity methods, such as molecular dynamics, provide detailed data, while empirical long-term aging studies inform changes in mechanical and electrical properties. Transfer learning further allows ML models to refine their predictions by incorporating degradation data. Together, these strategies can improve the reliability of material performance predictions. Physics-informed ML models enhance long-term reliability predictions by incorporating known degradation mechanisms like oxidation, hydrolysis, and thermal aging. This approach, combined with experimental data and computational simulations, improves model accuracy. Real-time monitoring through integrated sensors further enhances ML models by providing data on performance and degradation. AI analysis of this data facilitates predictive maintenance, identifying potential failures in polymer-based ESSs to extend device lifespan and ensure efficient operation.

Generative design frameworks, including VAEs and RL, are enhancing the optimization of polymer structures for long-term reliability. These models integrate degradation features, such as thermal and mechanical stress resistance, into the design process and enable multiobjective optimization to balance short-term performance and long-term reliability. To address the issue of data scarcity in polymer research, establishing large-scale aging data repositories will enhance the reliability of ML models in material analysis. Additionally, fostering collaboration among material scientists, chemists, and data scientists will lead to better degradation models and predictions regarding polymer longevity. Integrating accelerated aging data with multifidelity modeling in polymer-based energy storage systems boosts predictive accuracy and efficiency, reducing the need for extensive long-term testing. This approach not only accelerates the development of durable materials but also enhances sustainability by minimizing waste in energy storage technologies.

## 6. Future Directions

Future directions for the application of AI and ML in polymer discovery involve a range of innovative approaches aimed at enhancing efficiency and accuracy in materials science. Emphasis on the integration with experimental validation is crucial to ensuring that ML-generated polymer structures align with real-world performance, particularly concerning high molecular weight and polydispersity characteristics. Combining ML techniques with established physical and chemical theories can deepen the understanding of structure–property–performance relationships and improve model reliability. The development of smart material manufacturing platforms is also a priority, as these intelligent systems can automate the synthesis, characterization, and optimization of polymers, thus minimizing manual intervention. To address data scarcity, expanding datasets through theoretical data generation and collaboration on large-scale shared platforms will be essential. Advanced structural descriptors and improved representations of polymer structures, like BigSMILES and G-BigSMILES, facilitate accurate studies of polydispersity and long-chain characteristics. Additionally, adopting multiobjective optimization strategies is important for simultaneously enhancing various properties in materials. Generative design using ML models, such as variational autoencoders and graph neural networks, can uncover novel polymer structures with desirable traits. Incorporating prior theoretical knowledge into physics-informed models can enhance prediction accuracy while reducing reliance on extensive datasets. Focusing on functional and sustainable polymers through ML aligns with growing sustainability goals, while fostering interdisciplinary collaboration among polymer scientists, data scientists, and engineers can accelerate the discovery of next-generation materials. Collectively, these approaches aim to drive advancements in polymer science, significantly enhancing material discovery processes.

AI and ML can play a transformative role for sustainable polymer design and in optimizing their environmental impact. Life-Cycle Assessment (LCA) can benefit from AI-driven models that assess polymers from raw material extraction to disposal or recycling. By analyzing large datasets, ML can identify key factors affecting energy use, greenhouse gas emissions, and waste generation. This allows for scenario analysis to find the most sustainable production and recycling methods. Moreover, energy consumption in polymer synthesis can be enhanced through AI techniques like BO and RL, which help determine energy-efficient conditions. AI sensors allow for real-time monitoring and adjustments, while digital twins enable simulation of energy use to improve synthesis efficiency. AI enhances polymer recyclability by optimizing molecular structures for easier depolymerization and chemical recycling. ML models can predict polymer chain breakdown into reusable monomers by analyzing chemical compositions and degradation behavior. Generative design frameworks, like VAEs and GNNs, can be employed to develop biodegradable, chemically recyclable polymer structures, thereby minimizing environmental impact throughout their life cycle. Sustainable feedstock selection is an essential aspect of advancing environmentally friendly polymer synthesis. Utilizing biobased monomers from renewable sources, such as plant materials, decreases reliance on petroleum-based raw materials. AI helps identify and optimize these feedstocks, while ML supports waste valorization by exploring options like agricultural residues and plastic waste, promoting a circular economy. Multiobjective optimization is essential for sustainability, addressing trade-offs in energy efficiency, material performance, and environmental impact. AI and ML can assist in designing polymers that achieve a balance between high energy density, low synthesis energy, and recyclability. They also predict the environmental effects of polymer production, supporting informed material selection for greener options. AI enhances sustainability in polymer synthesis through real-time monitoring of energy use, waste, and emissions, allowing for dynamic process adjustments. Furthermore, AI optimizes recycling by analyzing polymer waste composition and suggesting efficient methods, promoting a sustainable approach to production and waste management.

Current strategies for integrating physical principles into ML for polymers involve PINNs and SR. PINNs enhance model accuracy by incorporating fundamental physical laws during training, improving predictions for properties like mechanical strength and thermal conductivity. In contrast, SR focuses on generating interpretable mathematical relationships between polymer structures and their properties, such as the correlation between molecular weight and viscosity. However, integrating polymer physics into ML faces challenges like the complexity of polymer behavior, scarcity of high-quality datasets, increased computational costs from incorporating physical principles, and the necessity of balancing data-driven and physics-based insights. Future research should develop hybrid models that merge PINNs with traditional ML, expand datasets to include detailed polymer dynamics, utilize symbolic regression for interpretable equations, and apply multifidelity learning to improve prediction reliability while reducing costs.

Future directions for the use of AI and ML in the field of polymers for energy storage suggest promising advancements in technology. One significant area is enhanced material design, where AI and ML can create novel polymer structures with optimized charge trap properties, high dielectric constants, and greater breakdown strengths. Additionally, predictive modeling can lead to the development of advanced ML models that forecast the long-term reliability, thermal stability, and energy storage efficiency of polymers under varying conditions. Integrating AI with quantum chemistry offers the potential to gain a deeper understanding of the electronic properties of polymers and their interactions with charge carriers. High-throughput screening powered by AI accelerates the discovery of high-performance materials, while data-driven insights allow for the analysis of experimental and simulation data to reveal hidden patterns that guide material optimization. Furthermore, ML can help identify environmentally friendly and cost-effective polymers, promoting the development of sustainable materials for energy storage applications. AI also plays a critical role in optimizing manufacturing processes, such as crosslinking and blending, thereby enhancing the scalability and performance of polymer-based capacitors. The development of multiscale modeling techniques will enable AI models to integrate molecular-level simulations with macroscopic performance predictions, providing a more comprehensive understanding of polymer behavior. Real-time monitoring using AI can improve operational safety by predicting failures in polymer energy storage systems. Finally, fostering interdisciplinary collaboration among material scientists, data scientists, and engineers will be essential to create robust AI frameworks tailored for advancing research in polymer energy storage.

## 7. Conclusions

This study presents a comprehensive literature review that highlights the application of ML and AI in polymer science, polymer design, characterization, and energy storage. It identifies various methods used in these technologies, evaluating their contributions to advancing research in these fields. However, the study also uncovers significant gaps, such as the limited availability of high-quality datasets for specific polymer types, challenges in capturing complex polymer structures, and issues of synthetic feasibility for AI-recommended polymers. In the realm of energy storage, it notes data scarcity for niche applications and difficulties in modeling intricate charge trap mechanisms. The analysis also examines the strengths and weaknesses of AI and ML; the strengths include accelerated discovery, efficient exploration of chemical spaces, and improved predictive modeling, while weaknesses involve dependencies on large datasets, high computing costs, and challenges in long-term reliability of polymer performance. Contributions of these technologies are significant, as they facilitate innovative polymer design and optimize properties in energy storage applications, utilizing generative models like variational autoencoders. Looking ahead, the study suggests future directions for polymer science, such as the expansion of datasets, development of advanced structural descriptors, and the integration of physical principles into ML models. For energy storage, it emphasizes enhanced material design and a focus on sustainable materials. Ultimately, the study underscores the transformative role of AI and ML in fostering efficient, sustainable, and innovative solutions in polymer science and energy storage, providing actionable insights and a roadmap for future advancements.

## Figures and Tables

**Table 1:** Advancements in Polymer Science: The Impact of Artificial Intelligence and Machine Learning on Polymer Design, Characterization, and Energy Storage Applications.

Topic	Methods	Key Insights and Contributions	References
Machine Learning Approaches in Polymer Science: Polymer Design and Characterization by AI-ML			
Development of a ML-based approach for the rapid characterization of SMPs.	Supervised ML, Multiscale Unsupervised Feature Selection Ensemble Learning, BRR, PNN, RBFN, RFN, AdaBoosting Random Subspace Method, Vision-Based Video Analysis	Rapid Characterization of SMP Behavior Data-Driven Modeling Feature Extraction from Thermal Videos Optimization of Material Properties Predictive Modeling for SMP Design Enabling Soft Robotics Applications Benchmarking and Validation AI/ML methods have advanced polymer design and characterization by enabling rapid and accurate modeling of SMP behavior, optimizing material properties, and supporting innovative applications like soft robotics.	Dutta et al. [44]
A novel graph-based representation and ML approach for predicting the properties of polymer molecular ensembles, addressing the challenges posed by their stochastic nature.	GNNs, D-MPNN, wD-MPNN, RF Fully Connected NN, Fingerprint Representations Sequence Sampling	Improved Property Prediction Representation of Molecular Ensembles Discrimination Between Polymer Variants Data Efficiency Support for Virtual Screening Application to Experimental Datasets Framework for Polymer Informatics These contributions enable faster and more accurate polymer design and characterization, advancing polymer informatics and supporting the discovery of novel materials for various applications.	Aldeghi and Coley [45]
The use of interpretable ML and physical descriptors to efficiently design and predict high thermal conductivity polymers for improved heat dissipation in organic electronics.	RF, XGBoost, MLP, SR SHAP Analysis, PCA, Mol2vec, BO	Efficient Prediction of TC Feature Engineering and Descriptor Optimization Interpretability of ML Models SR for Mathematical Modeling Virtual Screening of Polymer Databases Linking Hierarchical Structures to Thermal Properties Facilitating Experimental Design Integration of AI/ML methods has transformed polymer design from a trial-and-error approach to a systematic, data-driven framework. This accelerates the discovery of high-TC polymers and enhances understanding of structure-property relationships, driving innovation in polymer materials.	Huang et al. [46]
Utilizing ML algorithms to analyze the bead foam extrusion process of PLA, focusing on the impact of various processing parameters on bead foam density and melt pressure.	DTR, RF, GBR, LASSO Regressor, SVR, LR	Improved Prediction Accuracy Process Optimization Correlation Analysis Handling Complex Data Advancing Polymer Design Sustainability and Efficiency Feature Selection and Automation Scalability to Other Polymers Use of AI/ML enhances polymer science through accurate predictions, optimized processes, and the design of sustainable materials with tailored properties, representing a significant advancement in material science and engineering.	Shah et al. [47]
Optimization of low-density polyamide 12 foams using BO and ML techniques to enhance their properties and reduce experimental trials in the foaming process.	BO, AL Inverse Design ML Models: LR, DT, RF, GBR, GP, LASSO, SGDR, RR Python Framework	Optimization of Processing Parameters Reduction in Experimental Effort Inverse Design for Targeted Properties Enhanced Predictive Capabilities Insights into Process-Property Relationships Characterization of Foam Morphology Sustainability in Polymer Design Scalability and Future Applications AI/ML methods have transformed polymer design and characterization by optimizing processes, minimizing experimental effort, and enhancing understanding of process-property relationships. This leads to sustainable, high-performance polymer materials for various industrial applications.	Shah et al. [49]
Integration of AI and ML into polymerization and copolymerization processes to optimize synthesis, manufacturing, and material properties.	ML Algorithms: BO, SNOBFIT, ParEGO, TS-EMO Digital Twins, LLMs Data Analysis and Visualization: PCA, Correlation Matrices Simulation Techniques: CGMD, Kinetic Monte Carlo Simulations Python Scripting and APIs	Optimization of Polymerization Processes Enhanced Predictive Modeling Data-Driven Insights Accelerated Discovery Improved Characterization Scalability and Efficiency Design of Functional Polymers Integration of Theory and Experiment Future Potential AI/ML methods transform polymer design and characterization through precision control, predictive modeling, efficient data analysis, and faster discovery, advancing polymer science.	Advincula et al. [48]
Introducing “Polybot,” an AI-driven autonomous laboratory designed to optimize the solution processing of electronic polymer thin films, specifically focusing on PEDOT:PSS.	BO, GPR, Gaussian KDE, SHAP, LHS EI Acquisition Function RFR, UMAP	Efficient Exploration of Complex Parameter Spaces Accelerated Optimization of Polymer Properties Improved Data Quality and Reliability: Interpretability of Polymer Processing-Property Relationships Scalability and Practical Application Unbiased and Systematic Data Generation Advancing Polymer Characterization Generalizable Framework for Polymer Design Integration of AI/ML methods revolutionizes polymer design and characterization through high-throughput, data-driven optimization, enhancing insights into processing-property relationships and speeding up the development of high-performance materials. This represents a significant shift in materials science, leading to smarter, more efficient polymer manufacturing.	Wang et al. [50]
Artificial Intelligence Machine Learning Approaches for Polymers Energy Storage			
Developing a Ml-based framework to accelerate the design and discovery of polymer dielectrics.	KRR, GA	Accelerated Property Prediction Efficient Polymer Design Expansion of Polymer Options Guidance for Experimental Synthesis AI/ML methods have accelerated the discovery of advanced polymer dielectrics, meeting the demand for high-performance materials in energy storage.	Mannodi-Kanakkithodi et al. [58]
Using ML techniques to design and optimize the microstructure of polymer nanocomposites for enhanced energy storage applications.	RF, SVM, NN	Prediction of Energy Storage Density Optimization of Experimental Design Descriptor Weight Analysis Exploration of Effective Filler Structures Reliability Verification AI/ML methods have enhanced the design of polymer nanocomposites, boosting efficiency and innovation in energy storage materials.	Feng et al. [60]
The design of polymers for energy storage capacitors using ML and GA to identify candidates that can withstand high temperatures and electric fields.	ML Property Predictors, GPR models GA, Clamping Fitness Function, Duplication Checking, Chemical Screening Rules, FBS, Functional Group Screening Polymer Retrosynthesis Algorithm	Accelerated Polymer Discovery Targeted Property Optimization Enhanced Search Efficiency Synthetic Feasibility Assessment Customization for Specific Applications Public Accessibility AI/ML methods have streamlined polymer design, enhancing speed, efficiency, and precision in creating advanced materials for energy storage capacitors.	Kern et al. [59]
Optimization of data analysis for the design of PEMFC using ML algorithms to enhance performance and efficiency.	SVMR, LR, KNN	Optimization of PEMFC Performance Reduction of Computational Complexity Data-Driven Insights Validation Against Numerical Methods Scalability and Design Optimization AI/ML methods have greatly advanced the design, analysis, and optimization of PEMFCs, essential for energy storage and renewable energy systems.	Saco et al. [61]
Design of high-performance polymer membranes for organic solvent separations, utilizing a combination of ML, simulations, and experimental data to enhance solvent diffusivity predictions and identify optimal membranes.	MT PENN (PENN-1, PENN-2) GPR, NN Data Augmentation Polymer Genome Fingerprinting	Enhanced Predictive Accuracy Generalizability in Data-Limited Scenarios Physics-Based Modeling Screening Large Chemical Spaces Data-Driven Design The study on solvent separations showcases AI/ML methods that can also speed up the discovery and optimization of polymers for energy storage technologies like batteries, SCs, and dielectric materials.	Nistane et al. [64]
Development and optimization of polymer nanocomposites enhanced with carbon-based additives, specifically CNTs and CFs, for energy applications.	Hybrid Model Structure: combining RFR and SVR Dataset, Model Training and Validation Performance Metrics, Feature Importance Analysis Simulation and Design Optimization	Enhanced Predictive Accuracy Optimization of Material Properties Reduction in Experimental Effort Insights into Structure-Property Relationships Simulation of Untested Scenarios Improved Energy Storage Performance Error Mitigation and Data Quality Control Scalable Framework for Future Applications ML methods significantly enhance the design, performance, and development of polymer-based energy storage systems, improving their efficiency and scalability for practical use.	Chen et al. [62]
The discovery of LCPs with high TC using ML.	Supervised Learning, PU Learning High-Throughput Virtual Screening Descriptor Encoding, Optimization Clustering, Dimensionality Reduction	Predictive Modeling Data-Driven Design High-Throughput Screening Improved TC Molecular Orientation Analysis Phase Transition Insights ML enables the design of advanced polymers with high thermal conductivity, crucial for enhancing ESS performance and reliability.	Maeda et al. [63]

## Data Availability

No new data were created or analyzed in this study. Data sharing is not applicable to this article.

## Abbreviations

The following abbreviations are used in this manuscript:

AI	Artificial intelligence
AL	Active Learning
APIs	Application Programming Interfaces
BRR	Bayesian Ridge Regression
CGMD	Coarse-Grained Molecular Dynamics
CFs	Short-cut Carbon Fibers
CNTs	Carbon nanotubes
CPs	Conducting polymers
cryo-EM	Cryogenic electron microscopy
DCs	Dielectric capacitors
DFT	Density Functional Theory
DL	Deep learning
DT	Decision Tree
DTR	Decision Tree Regressor
D-MPNN	Directed Message Passing Neural Network
ECFP	Extended-Connectivity Fingerprints
EI	Expected Improvement
ELN	Ensemble Learner Network
ESDs	Energy storage devices
ESSs	Energy storage systems
FBS	Frequency-Based Selection
GA	Genetic algorithms
GAN	Generative Adversarial Networks
GB	Gradient Boosting
G-BigSMILES	Generative BigSMILES
GBR	Gradient Boosting Regressor
GNNs	Graph Neural Networks
GP	Gaussian Processes
GPR	Gaussian Process Regression
GPSR	Genetic Programming Symbolic Regression
IoT	Internet of Things
KDE	Kernel Density Estimation
KNN	K-Nearest Neighbor
KRR	Kernel Ridge Regression
LASSO	Least Absolute Shrinkage and Selection Operator
LCA	Life-Cycle Assessment
LCPs	Liquid crystalline polymers
LHS	Latin Hypercube Sampling
LIBs	Lithium-ion batteries
LLMs	Large Language Models
LR	Linear Regression
MDSs	Molecular Dynamics Simulations
ML	Machine learning
MLE	Mayo-Lewis Equation
MLP	Multilayer Perceptron
MSE	Mean squared error
MT	Multi-Task Learning
Mw	Molecular weight
MWD	Molecular weight distribution
NN	Neural Network
PA-12	Polyamide 12
PANI	Polyaniline
ParEGO	Pareto Efficient Global Optimization
PCA	Principal Component Analysis
PDMS	Polydimethylsiloxane
PEDOT	Poly(3,4-ethylenedioxythiophene)
PEDOT:PSS	Poly(3,4-ethylenedioxythiophene) doped with poly(4-styrenesulfonate)
PEMFC	Proton Exchange Membrane Fuel Cells
PENN	Physics-Enforced Neural Networks
PLA	Polylactic Acid
PNN	Probabilistic Neural Network
Ppy	Polypyrrole
PTh	Polythiophene
PU	Positive and Unlabeled
PVC	Polyvinyl chloride
R^2^	Coefficient of Determination
RBFN	Radial Basis Function Network
RF	Random Forest
RFN	Random Forest Network
RFR	Random Forest Regression
RL	Reinforcement Learning
RSM	Response Surface Methodology
RMSE	Root Mean Square Error
RR	Ridge Regression
sBO	Bayesian Optimization
SCs	Supercapacitors
SCIE	Science Citation Index Expanded (SCI-Expanded)
SEM	Scanning Electron Microscope
SGDR	Stochastic Gradient Descent Regression
SHAP	SHapley Additive exPlanations
SMILES	Simplified molecular-input line-entry system
SMPs	Shape Memory Polymers
SNOBFIT	Simplex and Stable Noisy Optimization by Branch and Fit
SPEs	Solid polymer electrolytes
SR	Symbolic Regression
SSB	Solid-state battery
SVR	Support Vector Regression
SVM	Support Vector Machines
SVMR	Support Vector Machine Regression
TC	Thermal conductivity
TS-EMO	Thompson Sampling Efficient Multi-Objective
UMAP	Uniform Manifold Approximation and Projection
UWG	Underwater Granulation
VAE	Variational Autoencoders
VFS	Virtual Forward Synthesis
wD-MPNN	Weighted directed message passing neural network
XGBoost	Extreme Gradient Boosting
